# The Effect of Two Different Insulin Formulations on Postprandial Hyperglycemia after High and Low Glycemic-Index Meal in Type 1 Diabetes

**DOI:** 10.3390/nu14163316

**Published:** 2022-08-12

**Authors:** Antonio Cutruzzolà, Martina Parise, Raffaella Fiorentino, Agata Romano, Viviana Molinaro, Agostino Gnasso, Sergio Di Molfetta, Concetta Irace

**Affiliations:** 1 Department of Clinical and Experimental Medicine, University Magna Graecia Catanzaro, 88100 Catanzaro, Italy; 2 Department of Health Science, University Magna Graecia Catanzaro, 88100 Catanzaro, Italy; 3 Diabetes Care Center, University Hospital Mater Domini, 88100 Catanzaro, Italy; 4 Medical School, University Magna Graecia Catanzaro, 88100 Catanzaro, Italy; 5 Section of Internal Medicine, Endocrinology, Andrology and Metabolic Diseases, Department of Emergency and Organ Transplantation, University of Bari Aldo Moro, 70121 Bari, Italy

**Keywords:** Faster Aspart, glycemic index, meal test, type 1 diabetes

## Abstract

Despite multiple pharmacological options, including rapid-acting insulin analogs, postprandial hyperglycemia is still highly prevalent in patients with type 1 and type 2 diabetes. We hypothesize that the new rapid-acting insulin formulation, the so-called faster-acting Aspart, may have a different effect in controlling postprandial hyperglycemic burden according to the quality of the meal compared to the traditional Aspart. Twenty-five patients with type 1 diabetes were consecutively recruited at the diabetes care center of the University Hospital affiliate of the Magna Græcia University of Catanzaro. Each patient performed four meal tests one week apart, two with a predefined high glycemic index (HGI) food and two with a low glycemic index (LGI) food using insulin Aspart once and Faster Aspart the other time. The 0–30 min, 0–60 min, and 0–120 min glucose Area Under the Curve (AUC) of postprandial glycemic excursion, calculated from continuous glucose monitoring data, were significantly lower with Faster Aspart administered before the HGI test meal as compared to Aspart. A significant difference in favor of Faster Aspart was also found when comparing the 0–60 min and 0–120 min AUC after the LGI meal. Faster Aspart may provide better postprandial glucose control than Aspart regardless of the glycemic index of the meal.

## 1. Introduction

Postprandial hyperglycemia is commonly expressed as punctual blood glucose measurements at 1–2 h after a meal [1,2,3] or as glucose Area Under the Curve (AUC) above the pre-prandial value in a 4-hour postprandial interval [1]. The international guidelines agree that patients with both type 1 and type 2 diabetes should test their postprandial blood glucose regularly, but a unique target level has not been identified yet [1,2,3]. Postprandial hyperglycemia greatly contributes to overall glucose control, possibly preventing people with diabetes from achieving glycated hemoglobin (HbA1c) targets and increases the risk of micro- and macrovascular complications [4,5,6,7,8]. Furthermore, postprandial hyperglycemia affects the quality of life and predicts pancreatic cancer and cognitive impairment [9,10,11,12].

Despite the availability of multiple pharmacological options, including rapid-acting insulin analogs, postprandial hyperglycemia is highly prevalent in patients with both type 1 diabetes and type 2 diabetes [13,14].

Many factors may influence the postprandial glycemic excursion, including the amount of ingested carbohydrates, the meal composition, the glycemic index (high and low) of the food, the regulation of gastric emptying, the substrate absorption, the proper suppression of hepatic glucose production during the meal, the inhibition of free fatty acid release, the pre-prandial glycemic value, and the pharmacokinetics of exogenous insulin [15,16]. High glycemic index (HGI) food is associated with fast carbohydrate absorption and may result in high blood glucose levels that occur soon after eating. In contrast, low glycemic index (LGI) food is slowly digested because of its fibers, protein, and fat content, with a more gradual increase in postprandial glucose [16]. Rapid-acting insulin analogs significantly reduce postprandial glycemic excursions and the risk of late postprandial hypoglycemia compared to human regular insulin and improve the quality of life [17]. In particular, insulin Aspart contains a single amino acid substitution (aspartic acid for proline) at position B28 compared to human insulin, which allows onset of action within 15 min and a peak at 30–90 min [18,19]. However, rapid-acting insulin analogs are still unable to reproduce the time-action profile of the endogenous insulin due to the inevitable time lag between the injection and the appearance in the systemic circulation [20,21,22]. Some practical strategies are commonly proposed to compensate for this time lag, such as anticipating the bolus injection by at least 20 min, splitting the bolus, and starting meals with fibers rather than carbohydrates.

In recent years, pharmacological research has attempted to resolve the gap between insulin injection and the onset of insulin action by implementing a new rapid-acting insulin formulation, the so-called faster-acting Aspart. It is a combination of conventional insulin Aspart with two excipients, the niacinamide, and the L-arginine, which enhance insulin absorption, in this way favoring an earlier onset of appearance and greater early pharmacokinetic and dynamic effect [23,24,25]. Faster Aspart is as effective as Aspart in improving HbA1c and fasting plasma glucose reduction and significantly reducing the postprandial glycemic excursion without increasing the risk of postprandial hypoglycemia [26,27,28,29,30].

We hypothesize that this new insulin formulation may have a different effect in controlling postprandial hyperglycemic burden according to the quality of the meal. Therefore, we have designed our study to evaluate the effectiveness of Faster Aspart on postprandial hyperglycemia, evaluated both through capillary blood glucose measurements and Continuous Glucose Monitoring (CGM), with two different test meals, one with HGI food and one with LGI food, in patients with type 1 diabetes.

## 2. Materials and Methods

The current research is an exploratory study with a cross-over design approved by the local Ethical Committee, Comitato Etico Regione Calabria Area Centro. Adult patients with type 1 diabetes using the calibration-free CGM system Dexcom G6 (Dexcom, Inc., San Diego, CA, USA) were consecutively recruited at the diabetes care center of the University Hospital affiliate of the Magna Græcia University of Catanzaro (Catanzaro, Italy). The inclusion and exclusion criteria of the study are reported in Table 1. Clinical characteristics and HbA1c values were collected from the electronic medical record.

The protocol was clearly illustrated to all eligible patients, and those who gave their written consent for participation in the study were enrolled. Each patient performed four tests one week apart, two on the HGI food and two on the LGI food, using insulin Aspart once and Faster Aspart the other time. All patients received detailed instructions on test meal preparation from an experienced dietician. In the two weeks before the trial tests, basal insulin dose, insulin:carbohydrate ratio (ICR), and insulin sensitivity factor (ISF) were optimized. The meals were prepared and consumed at home and contained the same amount of carbohydrates. The sequence of the type of insulin and the test meal were randomly assigned. The characteristics of the test meals are displayed in Table 2 and Table 3.

Participants were invited to consume the two test meals at dinner and measure capillary blood glucose before the meal. The test was permitted if the pre-meal blood glucose value was in the 70–160 mg/dL range. If blood glucose was out of the range, patients were advised to reschedule the test for the following day. They were also invited to perform each meal test at least 24 h after the sensor replacement. The Aspart and Faster Aspart insulin were injected before the meal as recommended by the summary of the product characteristics, and the doses were calculated according to the amount of carbohydrates and the ICR. On the days between the two meal tests, the patients were suggested not to change their behavior. All patients were given the Accu-Chek Guide blood glucose meter (Roche Diabetes Care, Indianapolis, IN, USA) to measure capillary glucose before the meal and 1-2-3-4 h after the meal. Interstitial glucose values measured by the sensor were collected from 5 min before meal initiation to 4 h after the meal through the Dexcom Clarity web-based platform (Dexcom, Inc., San Diego, CA, USA). Patients were finally invited to record any symptomatic hypoglycemic event occurring during the four hours after the meal test and to confirm the event by testing blood glucose.

The statistical analyses were performed by SPSS vers.25.0 (IBM, Armonk, NY, USA), and Graph PAD (San Diego, CA, USA). Normal distribution was assessed with the Shapiro–Wilk test. All variables were normally distributed. A two-way (within-within: time per insulin use interaction) repeated measures ANOVA (Analysis Of Variance) was used to compare the Faster Aspart versus Aspart effect on postprandial capillary blood glucose for both the HGI and LGI meal. We also assessed the glucose response at different intervals (0–30 min, 0–60 min, 0–120 min, and 0–4 h) after each meal and after Faster Aspart and Aspart by the incremental AUC trapezoid rule. The paired t-test was used to compare the interstitial glucose AUC 0–30 min, AUC 0–60 min, AUC 0–120 min, and AUC 0–4 h between the two rapid-acting insulin analogs and the two different meals. A two-tailed *p*-value < 0.05 was considered statistically significant for all the analyses. The sample size for the paired difference was calculated on an expected mean difference between baseline and 1-hour postprandial capillary blood glucose of 21 mg/dL between Faster Aspart and Aspart after the meal. The value refers to the difference reported in the ONSET-1 trial [25]. The standard deviation of blood glucose included in the formula calculating the sample size was preliminary calculated from a sample of 10 patients who performed the meal test with both insulin analogs and testing blood glucose with the meter used in the study. The number of subjects to be enrolled was 24 to achieve a power of 80% and a level of significance of 0.05. We recruited 25 patients in case of data loss or poor compliance of patients while performing the test meal.

## 3. Results

Characteristics of the 25 participants, 8 males and 17 females, have been reported in Table 4.

All patients completed the study for a total of 100 meal tests. HbA1c was at the target at the time of the enrollment, and all were injecting long-acting and rapid-acting insulin analogs. All patients completed the trial tests in the suggested time. The mean ICR was 14 ± 7 g/U, and the mean ISF was 49 ± 23 mg/dL. The mean total daily insulin dose was 54 ± 26 U, and the mean total bolus insulin dose was 21 ± 10 U. During the study period, there were no SARS-CoV-2 infections or overt COVID-19 cases among study participants.

**Figure 1:**
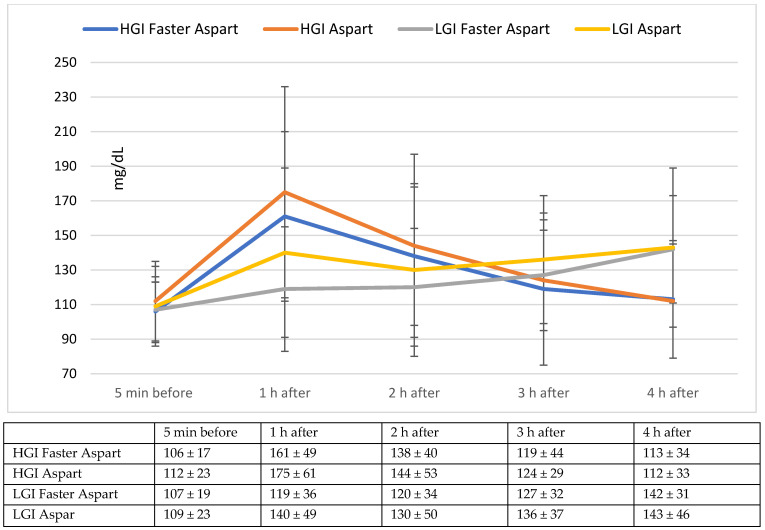
Blood glucose measurements (mean ± SD) 5 min before the meal and 1-2-3-4 h after the meal.

In Figure 1, we have illustrated the mean ± SD capillary blood glucose measured before the meal and 1-2-3-4 h after the meal. Mean pre-meal blood glucose was comparable in both trials before Faster Aspart and Aspart injection. No significant difference was detected when comparing postprandial blood glucose values after Faster Aspart and Aspart bolus injection during the HGI and LGI test meal.

No statistically significant difference was obtained when we compared the absolute postprandial increase in blood glucose with the two insulin analogs after HGI and LGI test meals (data not shown).

**Figure 2:**
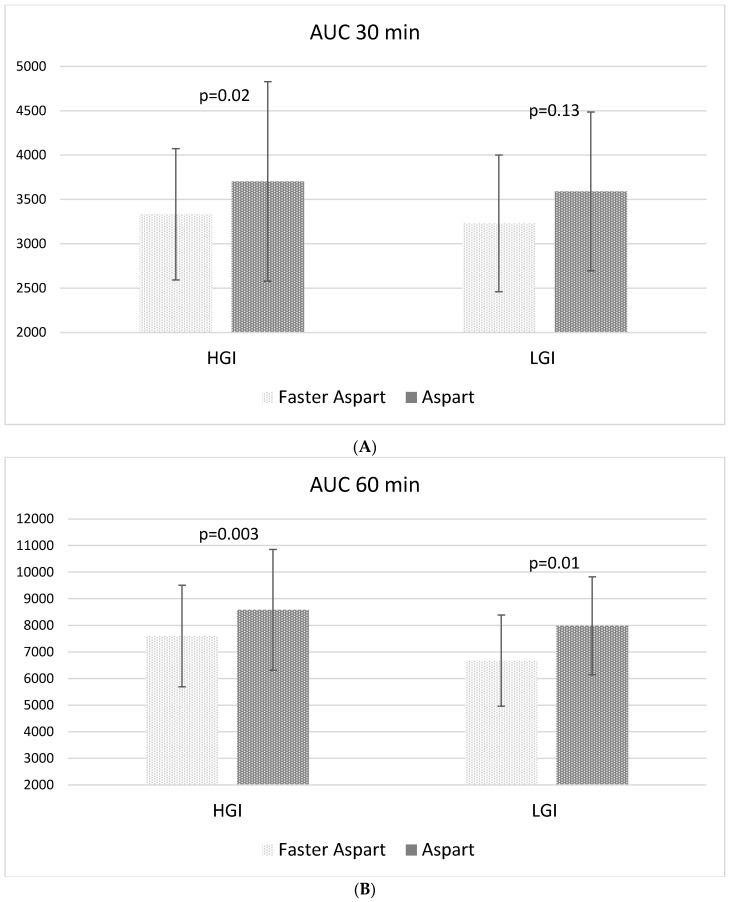

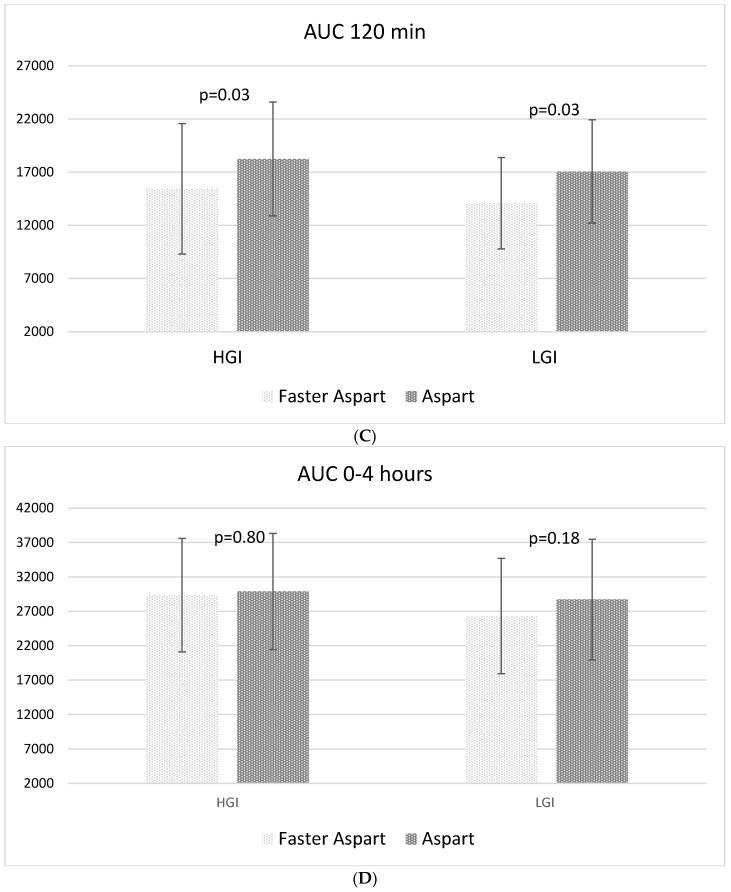
Glucose Area Under the Curve (AUC) in the (**A**) 30 min, (**B**) 60 min, (**C**) 120 min, and (**D**) 4 h following the meal tests.

Interestingly, the 0–30 min, 0–60 min, and 0–120 min AUC of postprandial glycemic excursion, calculated from CGM data, were significantly lower with Faster Aspart administered before the HGI test meal as compared to Aspart (Figure 2A–C). A statistically significant difference was also found between Faster Aspart and Aspart when comparing the 0–60 min and 0–120 min AUC after the LGI meal (Figure 2B, C). The overall postprandial AUC (0–4 h) was comparable between Faster Aspart and Aspart after the HGI and LGI trials (Figure 2D).

During the trial, no severe hypoglycemia occurred. Three episodes of symptomatic hypoglycemia were recorded during the HGI test meal, two after injection of Faster Aspart (15 min and 45 min), and one after Aspart (195 min). Five episodes of hypoglycemia were recorded during the LGI test meal, two after Faster Aspart (85 min, 120 min), and three after Aspart (65 min, 60 min, 60 min).

The AUC after two test meals and insulin analogs were also compared after excluding participants who had experienced hypoglycemia during the meal, and the results were the following: 0–30 min, AUC HGI Faster Aspart 3469 ± 725 vs. Aspart 3745 ± 1113, *p* = 0.05; 0–60 min, AUC HGI Faster Aspart 7727 ± 1955 vs. Aspart 8409 ± 2205, *p* = 0.03; 0–120 min, AUC HGI Faster Aspart 16126 ± 6812 vs. Aspart 17942 ± 4753, *p* = 0.18; 0–4 h, AUC HGI Faster Aspart 30202 ± 7921 vs. Aspart 29918 ± 8860, *p* = 0.91; 0–30 min, AUC LGI Faster Aspart 3415 ± 704 vs. Aspart 3553 ± 616, *p* = 0.44; 0–60 min, AUC LGI Faster Aspart 6976 ± 1598 vs. Aspart 8286 ± 1632, *p* = 0.01; 0–120 min, AUC LGI Faster Aspart 15570 ± 3015 vs. Aspart 17809 ± 4544, *p* = 0.08; 0–4 h, AUC LGI Faster Aspart 26594 ± 9117 vs. Aspart 29320 ± 9338, *p* = 0.17.

## 4. Discussion

The development of insulin formulations with different rates of absorption and action was prompted by the need to adequately control blood sugar levels, especially in the postprandial phase. The faster-acting insulin Aspart has an earlier onset of action than insulin Aspart, which should provide better control of the postprandial state without increasing the risk of hypoglycemia. In phase III trials, the efficacy of Faster Aspart has been evaluated with a standard liquid meal test and self-monitoring blood glucose [25,27,28,29,31,32,33,34].

To our knowledge, our study is the first to assess the effectiveness of Faster Aspart compared to Aspart after the ingestion of the two predefined HGI and LGI meals. Mealtime bolus injections of Faster Aspart resulted in better postprandial glucose control with both meals, as calculated from CGM data. Specifically, the AUC 0–30 min and 0–60 min following the HGI test meal and the AUC 0–60 min following the LGI test meal were significantly lower with Faster Aspart than Aspart in patients who did not experience postprandial hypoglycemia. The postprandial hyperglycemia, evaluated by the single glucose test 1 and 2 h after the HGI and LGI meals, was not statistically significant in our study, even if a difference was detected when the two insulin formulations were used. However, we can argue that the AUC better describes the overall postprandial hyperglycemic burden regardless of individual variability in carbohydrate absorption, gastric emptying, and meal composition. The intermittent or real-time use of CGM may help to manage postprandial hyperglycemia better and suggest a more effective insulin formulation. Indeed, to date, a definitive solution to prevent the occurrence of postprandial hyperglycemia after an HGI meal has not yet been found. Some nutritional strategies such as adding a moderate amount of protein, healthy fats, or dietary fiber may be implemented to reduce the effect of a carbohydrate-rich meal on postprandial hyperglycemia [35]. Also, management of bolus insulin doses for HGI meals remains a challenge, as traditional rapid-acting insulin analogs do not provide an acceptable glucose profile [36].

Given its rapid onset of appearance and greater early exposure compared to rapid-acting insulin analogs, Faster Aspart provides better postprandial glucose control and insulin dosing flexibility, with no need for injecting the bolus much in advance.

The pooled analyses evaluating the pharmacokinetic and pharmacodynamic characteristics of Faster Aspart in adults with type 1 diabetes reported an earlier onset of appearance (5 min) after injection of Faster Aspart compared to Aspart and two times greater early insulin exposure calculated as AUC0-30 min ratio Faster Aspart/Aspart [37]. According to the onset of action, the glucose-lowering effect, evaluated as the difference between capillary blood glucose and AUC before and after the meal test, was two times greater in the first 30 min. The total insulin exposure and the total glucose-lowering effect did not differ significantly, as well as the overall number of hypoglycemic events in the postprandial state. Our findings are in line with the pooled analysis, despite the absolute difference between the two analogs being less pronounced and the capillary blood glucose being not significantly different likely due to the different type of meal (liquid versus solid meal).

In some trials, Faster Aspart has been associated with a slightly increased risk of symptomatic hypoglycemia within the first two hours after a meal [25,27,29,32]. In our study, no severe hypoglycemia occurred, and non-severe symptomatic hypoglycemic episodes were infrequent following both test meals (HGI meal: 3/25, 12%; LGI meal: 5/25, 20%), with no differences between the two insulin analogs.

Given its shorter duration of action, another possible concern with Faster Aspart is the risk of late under-insulinization and hyperglycemia [23]. However, this was not an issue in our study, at least up to 4 h post-meal.

A major strength of our study design is that meal tests were randomized in their sequence and were all conducted at the same time of the day with no insulin on board. Moreover, CGM metrics were obtained from a measuring device approved as a substitute for capillary blood glucose in the period of best accuracy according to the manufacturer (i.e., starting from the second day after sensor placement) [38,39].

This study has some limitations. First of all, the test meals were performed at home; therefore, the investigators could not verify the strict observance of the study protocol. However, the trial was conducted during SARS-CoV-2 pandemic, and no alternative setting was feasible. Secondly, ISF was not accounted into the insulin bolus calculations. However, the mean ISF was 49 ± 23 mg/dL in the study population, and the test meal was rescheduled if pre-meal blood glucose was >160 mg/dL, so the non-inclusion of ISF did not substantially affect the final bolus insulin dose. Finally, the patients continued to use their usual insulin until the test meal, which may have influenced the results. Still, the clinical trials suggest that the three marketed rapid-acting insulin analogs—lispro, aspart, and glulisine—are equally efficacious [20].

SARS-CoV-2 breakthrough infection has occurred worldwide in recent months. Although there is no conclusive evidence that patients with diabetes are more vulnerable to the infection than the general population, multiple studies have shown that the coexistence of diabetes and COVID-19 is associated with poorer clinical outcomes, including increased mortality [40,41]. Consequently, achieving recommended glucose targets is strongly suggested during hospitalization due to COVID-19 [42]. Consistent with our results, by improving postprandial hyperglycemia, insulin Faster Aspart may help achieve glycemic targets during hospitalization in diabetic patients with COVID-19.

## 5. Conclusions

The results of our study demonstrate that insulin Faster Aspart is a valid alternative to Aspart to safely and effectively manage postprandial hyperglycemia in patients with type 1 diabetes, potentially providing more effective control of glycemic values in the three hours following a meal regardless of its glycemic index.

The effectiveness and safety of Faster Aspart should be further investigated in real-life studies with longer duration and/or including patients on insulin pump therapy. At the same time, predictors of the greater efficacy of Faster Aspart should be identified in order to tailor insulin treatment.

## Figures and Tables

**Table 1:** Inclusion and exclusion criteria.

Inclusion	Exclusion
Age > 18 years	Insulin pump therapy
Diagnosis of type 1 diabetes since at least one year	Pregnancy
HbA1c < 7.5% measured in the previous 2–3 months at the hospital laboratory	Coeliac disease
Multiple daily insulin injections therapy	Current infection
Stable insulin treatment for at least 3 months	Intense physical activity
Use of ICR for meal bolus calculation	Use of any drug interfering with glucose control
Use of Dexcom G6 CGM system	

ICR, insulin:carbohydrate ratio; CGM, continuous glucose monitoring.

**Table 2:** Composition of the two test meals.

HGI (CHO 70 g)	LGI (CHO 70 g)
White rice 40 g + frozen peas 120 g	Whole wheat pasta 80 g + parmesan cheese 5 g
Grilled lean beef 90 g	Grilled lean beef 90 g
Lettuce 50 g	Lettuce 50 g
EVOO 12 g	EVOO 12 g
Banana 180 g	Apple 100 g
Water 2 glasses	Water 2 glasses

HGI, high glycemic index; LGI, low glycemic index; CHO, carbohydrates; EVOO, extra virgin olive oil.

**Table 3:** Energy intake, macronutrients, and glycemic index of the two test meals.

	HGI	LGI
Energy (kcal)	522	542
Carbohydrates (g)	70	70
Total Fat (g)	15.2	17.3
Saturated Fat (g)	2.6	3.7
Proteins (g)	32	33.2
Fiber (g)	12	7.9
Glycemic index (%)	61	46

HGI, high glycemic index; LGI, low glycemic index.

**Table 4:** Characteristics of study participants.

Number	25
Age (years)	44 ± 16
Males (N/%)	8/32
Disease duration (years)	17 ± 11
Body weight (kg)	70 ± 14
BMI (kg/m^2^)	26 ± 5
HbA1c (%)	6.9 ± 0.6
Insulin (U/kg body weight)	0.6 ± 0.2

BMI, body mass index; HbA1c, glycated hemoglobin.

## Data Availability

The data presented in this study are available on request from the corresponding authors. Most of the data are sensitive and not publicly available.
